# A chimeric MERS-CoV virus-like particle vaccine protects mice against MERS-CoV challenge

**DOI:** 10.1186/s12985-022-01844-9

**Published:** 2022-06-27

**Authors:** Jung-Eun Park, Ji-Hee Kim, Jae-Yeon Park, Sung-Hoon Jun, Hyun-Jin Shin

**Affiliations:** 1 Laboratory of Veterinary Public Health, College of Veterinary Medicine, Chungnam National University, Daejeon, 34134 Republic of Korea; 2 Laboratory of Veterinary Infectious Diseases, College of Veterinary Medicine, Chungnam National University, Daejeon, 34134 Republic of Korea; 3 Research Institute of Veterinary Research, Chungnam National University, Daejeon, 34134 Republic of Korea; 4 Electron Microscopy & Spectroscopy Team, Korea Basic Science Institute, Cheongju, Chungcheongbukdo 28119 Republic of Korea

**Keywords:** MERS-CoV, Virus-like particle, Vaccine, Immunogenicity, Protective efficacy

## Abstract

**Background:**

Middle East respiratory syndrome coronavirus (MERS-CoV) causes severe respiratory disease in humans, with a case fatality rate of approximately 35%, thus posing a considerable threat to public health. The lack of approved vaccines or antivirals currently constitutes a barrier in controlling disease outbreaks and spread.

**Methods:**

In this study, using a mammalian expression system, which is advantageous for maintaining correct protein glycosylation patterns, we constructed chimeric MERS-CoV virus-like particles (VLPs) and determined their immunogenicity and protective efficacy in mice.

**Results:**

Western blot and cryo-electron microscopy analyses demonstrated that MERS-CoV VLPs were efficiently produced in cells co-transfected with MERS-CoV spike (S), envelope, membrane and murine hepatitis virus nucleocapsid genes. We examined their ability as a vaccine in a human dipeptidyl peptidase 4 knock-in C57BL/6 congenic mouse model. Mice immunized with MERS VLPs produced S-specific antibodies with virus neutralization activity. Furthermore, MERS-CoV VLP immunization provided complete protection against a lethal challenge with mouse-adapted MERS-CoV and improved virus clearance in the lung.

**Conclusions:**

Overall, these data demonstrate that MERS-CoV VLPs have excellent immunogenicity and represent a promising vaccine candidate.

## Background

Middle East respiratory syndrome (MERS) is a viral respiratory disease caused by a zoonotic coronavirus (MERS-CoV) that was first identified in Saudi Arabia in 2012 [1]. Bats are likely the original reservoirs, and coronaviruses similar to MERS-CoV have been identified in bats [2]. Zoonotic transmission from dromedary camels (the host reservoir for this virus) along with human-to-human transmission are the two known modes of infection [3–5]. MERS provokes a high mortality rate (~ 35%) associated with severe lung diseases such as acute respiratory distress syndrome and pneumonia [4, 6, 7]. To date, MERS has been reported in 27 countries and caused 888 deaths in 2,578 confirmed cases (October 2021, World Health Organization [WHO]), and MERS-CoV infections are still occurring in the Middle East. However, there are no approved vaccines or treatments for MERS in humans or animals.

MERS-CoV, a betacoronavirus, is an enveloped virus containing positive-sense single-stranded RNA. The viral genome encodes two large nonstructural proteins (ORF1a and ORF1b), four structural proteins (spike [S], envelope [E], membrane [M], and nucleocapsid [N]), and accessory proteins [8]. The S protein mediates virus entry and induces the production of neutralizing antibodies [9]. The M and E proteins are necessary for the formation of virus particles [10, 11]. N proteins form helical ribonucleoprotein complexes with viral RNA [12].

Many vaccine candidates are under development, including those based on viral vectors, nanoparticles, DNA, and subunit vaccines [13, 14]. However, none of these vaccines have been approved for human use, and research is ongoing to produce an effective and safe vaccine that can prevent MERS-CoV infection. Virus-like particles (VLPs) are considered an attractive platform for viral vaccines because of their high immunogenicity and safety [15–17]. Studies have produced VLPs in insect cells transfected with E and M structural proteins [11], which are essential for CoV VLP production [10, 18]. Recent publications have also shown that murine hepatitis virus (MHV) N proteins are more capable of supporting VLP formation than MERS N proteins [19, 20]. Although MERS-CoV VLPs produced from insect cells expressing the E, M, and S proteins have been shown to induce specific humoral and cellular immunity in rhesus macaques, their protective efficacy has not been tested [11]. In this study, we generated MERS-CoV VLPs in a mammalian expression system and evaluated the immunogenicity and protective efficacy of MERS-CoV VLPs in a mouse model.

## Methods

### Cells and viruses

HEK293 (KTCC 21,573) cells were maintained in Dulbecco's modified Eagle’s medium (DMEM, HyClone) supplemented with 10% (vol/vol) fetal bovine serum (FBS, HyClone), 10 mM HEPES, 100 mM sodium pyruvate, 0.1 mM nonessential amino acids, 100 U/ml penicillin G and 100 μg/ml streptomycin. Huh7 (KTCC 60,104) and Vero (KTCC 10,081) cells were maintained in DMEM supplemented with 10% (vol/vol) FBS, 100 U/ml penicillin G and 100 μg/ml streptomycin. Cell culture materials and reagents were obtained from SPL and Gibco. All cells were maintained at 37 °C in 5% CO_2_.

Mouse-adapted (MA) MERS-CoV was provided by Dr. Paul McCray at the University of Iowa and propagated in Vero cells as described previously [21].

### Plasmids

Human codon-optimized sequences of genes encoding the S, M, E, and N-myc tag proteins of MERS-CoV (GenBank: NC019843) were synthesized by GenScript Biotechnology. The genes were cloned into the expression vector pcDNA3.1 via the *EcoRI* and *XbaI* restriction sites. A gene encoding the MHV N protein with a myc tag was amplified with specific primers (forward: CCGAGCTCTTTAAGGATGTCTTTTGT, reverse: CCGCTCGAGTTACAGATCCTCTTCTGAGATGAGTTTTTGTTCCACATTAGAGTC) and cloned into the pCAGGS vector via the *SacI* and *XhoI* restriction sites. pNL4.3-Luc R- E- was obtained from the NIH AIDS Research and Reference Program, cat. # 3418.

### Generation and concentration of chimeric MERS-CoV VLPs

HEK293 cells were transfected with plasmids encoding the indicated structural proteins individually or in combination using polyethylenimine. Supernatants containing VLPs were collected 48–72 h post-transfection and then concentrated 100-fold by centrifugation at 100,000 × g at 4 °C for 2 h through a 20% sucrose cushion. The concentrated VLPs were suspended in phosphate-buffered saline (PBS). The total protein concentrations in the VLPs were measured using a Pierce™ BCA Protein Assay Kit (Pierce Biotechnology) according to the manufacturer’s instructions. VLPs were stored at − 80 °C until use.

### Western blot analysis

Transfected cells were lysed in Triton X-100 lysis buffer (1% Triton X-100, 50 mM Tris–Cl [pH 8.0], 150 mM NaCl, 1 mM EDTA) on ice and cleared by centrifugation at 1,000 × g for 10 min at 4 °C. The concentrated VLPs and transfected cell lysates were mixed with SDS solubilizer. Samples were heated at 95 °C for 10 min, separated in 8% or 15% (wt/vol) polyacrylamide-SDS gels, transferred to PVDF membranes, and probed with polyclonal mouse anti-MERS-CoV S (Sino Biological, 1:1000), polyclonal rabbit anti-MERS-CoV M (GeneTex, 1:1000), polyclonal rabbit anti-MERS-CoV E (GeneTex, 1:1000), or monoclonal mouse anti-myc (Santa Cruz, 1:1000) antibodies. The membranes were then probed with horseradish peroxidase (HRP)-conjugated goat anti-mouse IgG (Promega, 1:1000) or anti-rabbit IgG (Bioss, 1:1000) and incubated with ECL substrate (Thermo Fisher Scientific), and the signals were detected using a Fusion Solo X (Vilber). Band density on western blot membranes was analyzed using Evolution Capt software.

### Cryo-electron microscopy (Cryo-EM)

Three microliters of MERS-CoV VLPs at 100 µg/ml or a negative control (concentrated supernatants from S-transfected cells) were loaded onto freshly glow-discharged UltrAuFoil R1.2/1.3 300-mesh grids (Quantifoil Micro Tools GmbH), followed by plunge freezing using a Vitrobot Mark IV (Thermo Fisher Scientific Inc.) at 100% relative humidity and 4 °C. Image data collection was performed using a Talos Arctica G2 transmission electron microscope (Thermo Fisher Scientific Inc.) at the Korea Basic Science Institute operating at a 200 kV acceleration voltage under parallel illumination conditions. Images were acquired in EFTEM mode with a slit width of 20 eV using a BioQuantum energy filter and a K3 direct electron detector (Gatan Inc.) at a nominal magnification of 100,000 × corresponding to a calibrated pixel size of 0.83 Å per pixel with spot size 2 and 50 µm C2 aperture. Data collection was performed using EPU software (Thermo Fisher Scientific Inc.), and exposures were recorded at a defocus range of − 1.5 µm in electron counting mode for 3.0 s at a dose rate of 11.6 e-/pixel/s, resulting in a total dose of 50 e-/Å2.

### Mouse immunization, challenge, and sample collection

The animal experiments were performed according to the protocol approved by the Institutional Animal Care and Use Committee of Chungnam National University (2019012A-CNU-188). All work with MA MERS-CoV was performed in an animal biosafety level 3 laboratory of Jeonbuk National University.

Human dipeptidyl peptidase-4 (hDPP4) knock-in (KI) mice were provided by Dr. Paul McCray at the University of Iowa (Iowa City, IA). Eight- to ten-week-old C57BL/6 (Samtako, South Korea) and hDPP4 KI mice were randomized into three groups of eight mice each. Mice were immunized with 10 μg of MERS-CoV VLPs or PBS. Antigens were mixed 1:1 (vol/vol) with alum adjuvant (Sigma) and delivered to mice intramuscularly in the gastrocnemius muscle twice at a 2-week interval. Serum samples were collected from the retro-orbital plexus at 28 days post-immunization.

For virus challenge, at 28 days post-immunization, immunized mice were infected intranasally with 10^4^ PFU of MA MERS-CoV in 50 μl of DMEM. Mice were examined daily, and weights were recorded. At 3 days post-challenge, 3 mice were sacrificed, and lung tissues were harvested for virus titration and histopathological analysis.

### Pseudovirus neutralization test

A standard microneutralization assay was used to determine the levels of neutralizing antibodies against pseudotyped HIVluc-MERS virus. The pseudotyped HIVluc-MERS virus was prepared as described previously [22]. The relative 50% neutralizing titer (NT_50_) was defined as the highest serum dilution that exhibited a 50% decrease in luciferase activity compared to that of the negative control.

### Enzyme-linked immunosorbent assay (ELISA)

ELISA plates were pre-coatedprecoated overnight with pseudotyped HIVluc-MERS virus at 4 °C. The coated plates were washed three times with PBS-T (PBS containing 0.1% Tween 20) and then blocked with blocking buffer (5% skim milk in PBS-T) for 2 h at 25 °C. Plates were incubated with the twofold serial dilution of mouse serum starting from 1:100 and incubated at 25 °C for 2 h. Then, the plates were washed four times with PBS-T and incubated with HRP-conjugated anti-mouse IgG (Bethyl Laboratories, 1:1000), IgG1 (Bethyl Laboratories, 1:1000), or IgG2a (Bethyl Laboratories, 1:1000) at 25 °C for 1 h. After four washes with PBS-T, the plates were incubated with a 0.03% 3,3′,5,5′-tetramethylbenzidine solution (Koma Biotech) for 15 min at 25 °C. The reaction was stopped with 1 N H_2_SO_4_. The optical density (OD) at a wavelength of 450 nm was measured using an ELISA plate reader (Bio-Rad). The endpoint titer was defined as the last serum dilution at which the OD value was above the average of negative-control wells (secondary antibody only).

### Virus titration

Lungs were homogenized in PBS using a Precellys Evolution tissue homogenizer (Bertin Technologies). Viral titers were determined in Vero cells using a plaque assay as described previously [23]. Vero cells prepared in 12-well plates were infected with tenfold serially diluted lung homogenates for 1 h at 37 °C. After 1 h, the inoculums were aspirated, and the cells were kept for 72 h at 37 °C in MEM containing 0.8% agar and 2% FBS. Infected cells were fixed in 10% formaldehyde and stained with 0.1% crystal violet to delineate plaques.

### Histopathological analysis

Histopathological analysis was performed as described previously [24]. Lungs were fixed in 10% formalin overnight and then embedded in paraffin. Lung sections (~ 4 µm thick) were stained with hematoxylin and eosin. Lungs were scored for edema, hyaline membrane formation, cellular debris (neutrophils), and hemorrhage, with scores of 0, 1, 2, 3, and 4 representing detection in 0%, less than 5%, 6% to 33%, 33% to 66%, and more than 66% of lung fields, respectively.

### Statistical analysis

All experiments were independently repeated at least two times. Data are presented as the mean ± SD. Statistical significance was calculated using the two-tailed unpaired Student’s t test procedure. A P value < 0.05 was considered statistically significant.

## Results

### Construction of MERS-CoV VLPs in eukaryotic cells

**Fig. 1:**
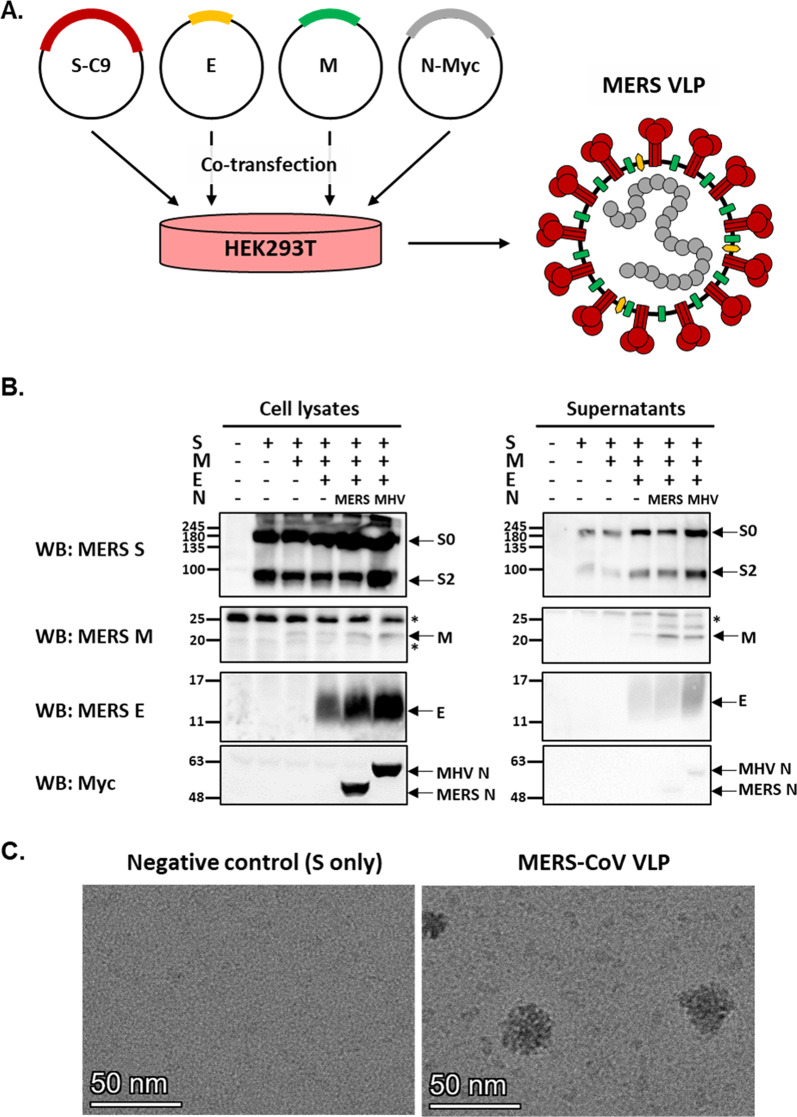
Generation and characterization of chimeric MERS-CoV VLPs. **A** Schematic outline of MERS-CoV VLP production. Plasmids encoding the structural proteins S, E, and M and N-myc were transfected into HEK293T cells. VLPs were collected from culture medium between 48 and 72 h. **B** Western blot images depict structural proteins in cell lysates of transfected cells (left) and concentrated VLPs (right). The numbers on the left indicate molecular mass in kilodaltons. S, E, M, and N proteins are depicted. The asterisk (*) indicates non-specific binding. **C** Cryo-EM images of a chimeric VLP and negative control (S only). Bars = 50 nm

CoV VLPs are produced upon co-expression of M, E, and N in a mammalian expression system [25]. For the formation of MERS-CoV VLPs, we co-expressed the S, E, M, and N structural proteins in HEK293 cells and collected the supernatant at 48–72 h post-transfection (Fig. 1A). The expression of structural proteins in cell lysates and purified VLPs (supernatants) was examined by western blotting (Fig. 1B). As expected, S-specific bands were shifted up from the expected size of 149 kDa due to glycosylation. Small amounts of secreted S proteins were detected in the supernatants of cells transfected with the S or S and M genes. S secretion slightly increased by 2- (S0 fragment) to fourfold (S2 fragment) when E and N genes were co-transfected. Consistent with previous reports, MHV N was more capable of supporting MERS-CoV VLPs, providing up to 80% VLP production relative to that with MERS N. We next investigated the morphology of VLPs by cryo-EM. Cryo-EM analysis revealed that the average diameter of MERS-CoV VLPs was approximately 30 nm (Fig. 1C). MERS-CoV VLPs were not present in the supernatants from cells transfected with S (Fig. 1C, negative control). These results indicated that MERS-CoV VLPs were produced in mammalian cells expressing E, M, and MHV N structural proteins.

### Immune responses to chimeric MERS-CoV VLPs in immunized mice

**Fig. 2:**
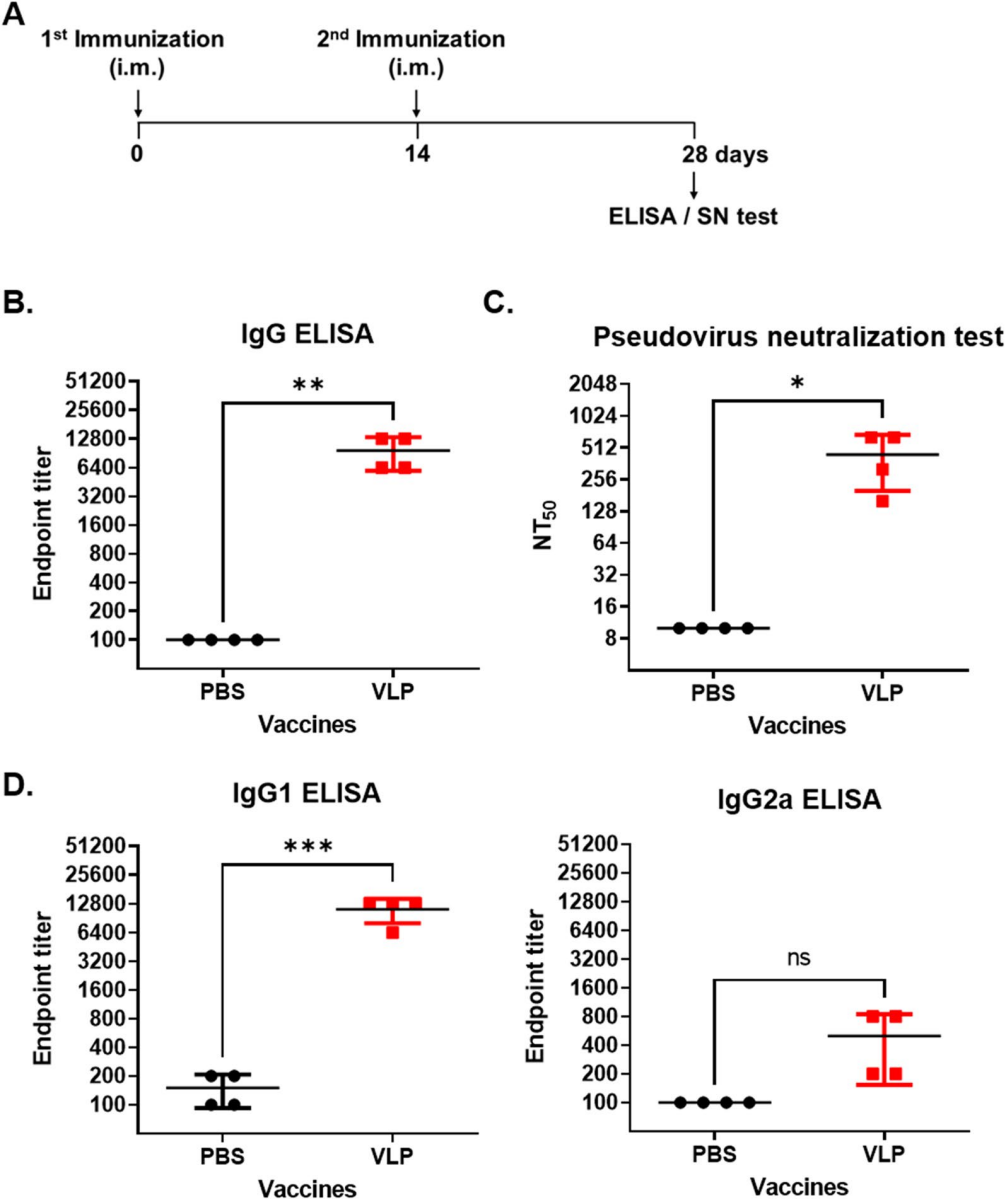
Chimeric MERS-CoV VLP immunization induced the production of S-specific IgG and neutralizing antibodies. **A** Schematic diagram of the immunization protocol. C57BL/6 mice were intramuscularly immunized with chimeric MERS-CoV VLPs or PBS. At 28 days post-immunization, serum samples were collected. **B** Endpoint titers of MERS S-specific IgG were measured by ELISA. **C** Detection of neutralizing antibody titers in the serum of the immunized mice using a pseudovirus neutralization test. The results are expressed as the mean (n = 4) NT_50_ ± SD. **D** Endpoint titers of MERS S-specific IgG1 and IgG2a were measured by ELISA. Statistical significance was assessed using Student’s t test. *, P < 0.05, **, P < 0.01; ***, P < 0.001; ns, not significant

To examine the immunogenicity of MERS-CoV VLPs in mice, C57BL/6 mice were intramuscularly immunized with MERS-CoV VLPs twice at a two-week interval (Fig. 2A). At 4 weeks after immunization, serum IgG titers were examined by ELISA. The IgG titers in mice immunized with MERS-CoV VLPs were significantly higher than those in mice immunized with PBS (Fig. 2B). To further determine the protective efficacy of MERS-CoV VLP immunization, the neutralizing activity of serum samples was analyzed using a virus neutralization assay. The average NT_50_ titer in mice immunized with MERS-CoV VLPs was 440 ± 240 (Fig. 2C). These results indicated that MERS-CoV VLP immunization induced strong immune responses with virus-neutralizing properties in mice.

To determine whether Th1 and/or Th2 immune responses were induced by vaccination, the levels of specific IgG1 and IgG2a subclasses were measured. VLP immunization induced the production of antigen-specific IgG1 but not IgG2a (Fig. 2D). These results indicated that MERS-CoV VLP immunization induced a Th2-biased immune response in mice.

### Protective efficacy of immunization with chimeric MERS-CoV VLPs in mice

**Fig. 3:**
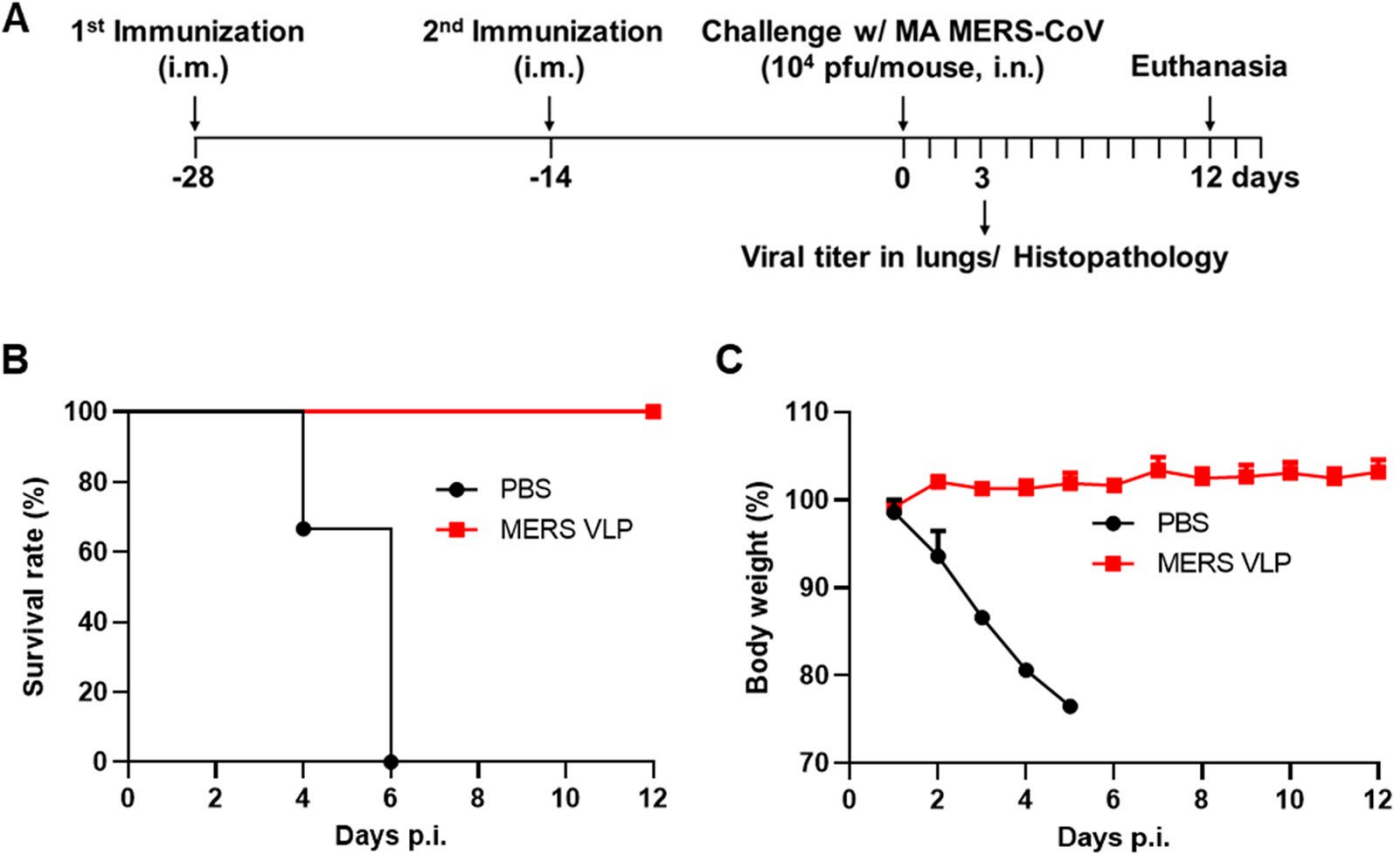
Chimeric MERS-CoV VLP immunization completely protects hDPP4 KI mice from lethal MERS-CoV challenge. **A** Schematic timeline of the immunization and challenge protocol. hDPP4 KI mice were intranasally immunized with chimeric MERS-CoV VLPs or PBS. At 28 days post-immunization, the mice were intranasally infected with 10^4^ PFU of MA MERS-CoV. **B** Survival was monitored daily for 12 days. **C** Weight loss was monitored daily for 12 days and is presented as relative to initial body weight. The results are expressed as the mean (n = 5) ± SEM values and are representative of at least two independent experiments

To determine the protective efficacy of MERS-CoV VLP immunization against a lethal virus challenge, hDPP4 KI mice were immunized with MERS-CoV VLPs. At 28 days after immunization, the mice were intranasally challenged with MA MERS-CoV (Fig. 3A). PBS-immunized mice gradually lost body weight following MA MERS-CoV challenge, and they all died (Fig. 3B and C). In contrast, MERS-CoV VLP-immunized mice survived up to 12 days and did not show weight loss (Fig. 3B and C), indicating that MERS-CoV VLPs protected mice against lethal challenge.

**Fig. 4:**
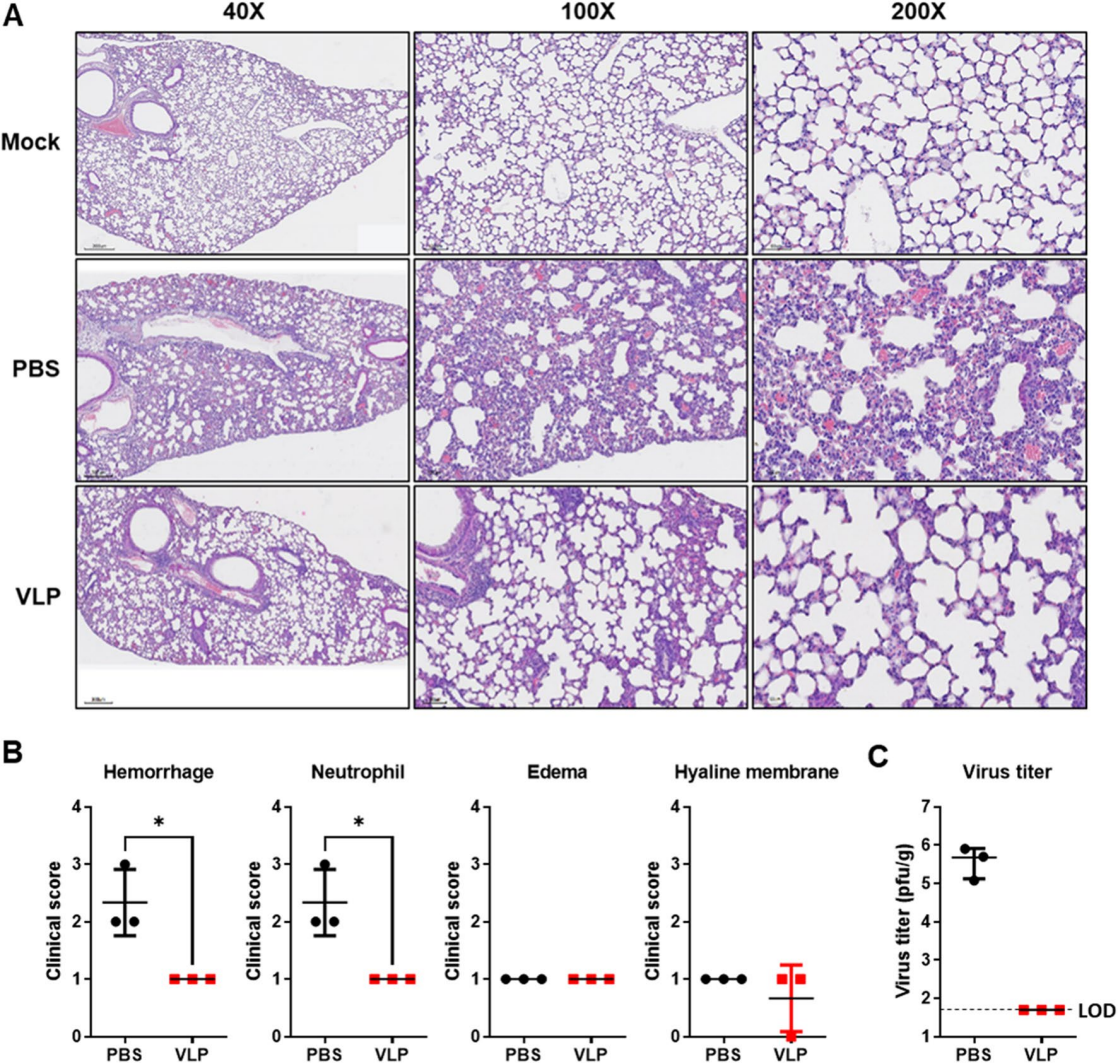
Chimeric MERS-CoV VLP immunization reduced histopathological changes and virus titers in the lung. **A** Representative images of H&E-stained lung sections from chimeric MERS-CoV VLP-immunized, PBS-immunized, or mock-infected hDPP4 KI mice at 3 days after MA MERS-CoV challenge. **B** Summary scores for disease in the lung sections. n = 3 mice/group. **C** At 3 days after virus challenge, virus lung titers were quantified using a plaque assay. The results are expressed as the mean (n = 3) ± SD. LOD, limit of detection. Statistical significance was assessed using Student’s t test. *, P < 0.05

Histopathology analysis of lungs after challenge with MA MERS-CoV indicated that MA MERS-CoV increased the presence of cellular debris, edema, and hyaline membranes (Fig. 4A). MERS-CoV VLP-immunized mice exhibited less evidence of lesions indicative of severe disease and significantly reduced hemorrhage and neutrophil infiltration (Fig. 4B). To determine whether immunization provides sterilizing immunity in the lung, virus titers in the lung were measured (Fig. 4C). Lung virus titers in PBS-immunized mice were approximately 5 × 10^5^ pfu/g. No virus was detected in MERS-CoV VLP-immunized mice, indicating that MERS-CoV VLPs resolved infection from the lungs.

## Discussion

VLP-based vaccines, particularly those produced via the baculovirus expression system in insect cells, are widely used for vaccine development, and several human vaccines using VLPs have been approved by the FDA [26, 27]. For CoV vaccines, severe acute respiratory syndrome (SARS)-CoV VLPs have been developed, and their induced immune responses and protective efficacy have been determined [20]. Wang et al. reported that MERS-CoV VLPs produced in insect cells induce specific humoral and cellular immunity in rhesus macaques, although the protective efficacy was not tested [11]. In the present study, we evaluated the efficacy of immunization of mice with chimeric VLPs for protection against MERS-CoV infection. Here, we demonstrated that chimeric VLPs formed in a mammalian expression system were immunogenic and provided protective immunity against lethal infection. Although various strategies for MERS-CoV vaccines, including recombinant S proteins, inactivated virus, DNA vaccines and other approaches, have been reported, to our knowledge, this is the first report testing chimeric VLPs propagated in mammalian cells as MERS-CoV vaccine candidates and evaluating vaccine efficacy in an in vivo model.

Previous findings showed that MERS-CoV VLPs could be produced by the coinfection of insect cells with baculoviruses expressing the S, M and E genes [11]. However, the expression of these three proteins (MERS-CoV S, M and E) or even four proteins (MERS-CoV S, M, E, and N) did not result in efficient VLP formation in HEK293 cells (Fig. 1B). A recent study demonstrated that SARS-CoV-2 VLP production was dependent on N proteins and that the carboxy-terminal domains of the N protein are critical for virus particle assembly [19]. Consistent with previous findings, we demonstrated that VLP formation was facilitated in the presence of MHV N (Fig. 1B). These data suggest that N proteins are important for CoV assembly and VLP formation in mammalian cells.

High S-specific IgG titers (Fig. 2B) and IgG1/IgG2a ratios (Fig. 2D) were detected in mice immunized with chimeric MERS-CoV VLPs, indicating that chimeric MERS-CoV VLP immunization induces Th2-biased humoral immune responses. Previous observations suggest that antibodies against CoV S proteins generally provide protective immunity [28, 29]. However, in the case of feline infectious peritonitis virus, S-specific antibodies fail to block virus infection and augment virus replication through antibody-dependent enhancement [30–32]. In our study, the antibodies induced by VLP immunization readily neutralized MERS-CoV entry in vitro, with neutralizing titers of 440 ± 240 (Fig. 2C). This titer is 10 times higher than that of MERS-CoV VLPs produced in insect cells co-expressing M, E, and S proteins [11]. More importantly, all mice immunized with chimeric MERS-CoV VLPs survived after virus challenge (Fig. 3B) and showed virus clearance in the lung (Fig. 4C). These results indicate that humoral immunity induced by chimeric MERS-CoV VLP immunization can provide complete protection against lethal virus infection. The humoral immune response mediated by B cell-derived neutralizing antibodies is powerful in eradicating viral infections, but recent studies have shown that specific antibodies may be maintained for only 2 months [33]. Accumulating evidence suggests that cellular immunity mediated by T cells and memory B cells is important for prolonging vaccine efficacy [34, 35]. Therefore, it will be necessary to investigate how MERS-CoV VLP immunization elicits memory B cell production as well as cellular immune responses.

The advantage of VLPs in mammalian cells is that they carry precise post-translational modifications that are important for immune responses and protection. However, unless the challenges of large-scale production and purification are addressed, the use of VLP vaccines produced in mammalian cells will be limited. Even if proof-of-concept has been demonstrated, there is a need to improve VLP production, such as by developing cells that stably express structural proteins.

## Conclusions

Here, we showed that a VLP-based vaccine is sufficient to provide protective immunity against lethal challenge. The safety of VLP-based vaccines has already been confirmed with commercially available vaccines. These results suggest that a VLP-based vaccine is a promising vaccine platform for CoV infection. The discovery that MERS-CoV VLPs protected mice against lethal MERS-CoV challenge suggests their potential use as a vaccine platform for emerging viruses such SARS-CoV-2.

## Data Availability

All datasets in the current study are available from the first authors upon reasonable request.

## Abbreviations

MERS: Middle East respiratory syndrome
CoV: Coronavirus
VLP: Virus-like particle
S: Spike
E: Envelope
M: Membrane
N: Nucleocapsid
DMEM: Dulbecco: s modified Eagle: s medium
FBS: Fetal bovine serum
MA: Mouse-adapted
MHV: Murine hepatitis virus
PBS: Phosphate-buffered saline
HRP: Horseradish peroxidase
hDPP4: Human dipeptidyl peptidase-4
KI: Knock-in
PFU: Plaque-forming units
SN: Serum neutralization
ELISA: Enzyme-linked immunosorbent assay
EM: Electron microscopy
OD: Optical density
